# Project PIX (Post Intensive care eXercise): impact on physical fitness and focus group analysis of quality of life following exercise rehabilitation

**DOI:** 10.1186/cc12472

**Published:** 2013-03-19

**Authors:** J Goodman, W Walker, J Wright, G Danjoux, S Howell, D Martin, S Bonner

**Affiliations:** 1 James Cook University Hospital, Middlesbrough, UK; 2 West Park Hospital, Darlington, UK; 3 University of Leeds, UK; 4 Teeside University, Middlesbrough, UK

## Introduction

This study assessed the effect of a hospital-based aerobic exercise programme on physical fitness and health-related quality of life (HRQoL) for survivors of ICU admission. Including the qualitative arm of the study, we examined the patient experience after critical illness, their views of the exercise programme and the effects on their HRQoL.

## Methods

A randomised controlled trial was undertaken in adult survivors of ICU admission. They were allocated to receive an 8-week in-hospital supervised aerobic programme consisting of two cycle ergometry and one unsupervised session per week (exercise group) or no exercise (control group). Primary outcomes were the anaerobic threshold (in ml O_2_/kg mass/minute), physical function and mental health scores (SF-36 questionnaire), measured at weeks 9 and 26. Participants were then allocated to focus groups where the interpretation of experiences was compared with outcomes from the PIX study.

## Results

Fifty-nine patients were recruited to the study. The anaerobic threshold increased at week 9 in the exercise group by a clinically and statistically significant amount of 2 ml O_2_/kg mass/minute (90% CI, 1 to 3 ml/kg/minute). There was further improvement in fitness levels in both groups by week 26 (although no significant difference between groups). No significant difference in HRQOL measures between groups was demonstrated; however, the exercise group did show an improvement in their mental health scores. The focus groups centred on feelings of isolation, abandonment, vulnerability, dependency and reduced physical activity post hospital discharge. Many reported a lack of social inclusion as they did not have the energy or confidence to venture outside. However, those in the exercise group felt that the rehabilitation programme was motivating, built up confidence, improved fitness, helped social interaction and gave them a sense of achievement.

## Conclusion

The 8-week exercise intervention resulted in statistically significant improvements in fitness at 9 weeks while focus group participants highlighted the positive effects of the exercise intervention leading to enhanced energy levels, motivation and achievement. Psychological benefits of the exercise programme are apparent from the focus group, emphasising the important link between physical and mental health.

